# External ureteric stent versus internal double J stent in kidney transplantation: a retrospective analysis on the incidence of urological complications and urinary tract infections

**DOI:** 10.3389/fneph.2023.1130672

**Published:** 2023-05-16

**Authors:** Ietje T. Hazenberg, Stephanie J. M. Middelkoop, Anoek A. E. de Joode, Juliette D. Rabbeljee, Robert A. Pol, Benjamin H. J. Doornweerd, Jan-Stephan F. Sanders, Coen A. Stegeman

**Affiliations:** ^1^ Department of Internal Medicine, Division of Nephrology, University Medical Center Groningen, University of Groningen, Groningen, Netherlands; ^2^ Department of Surgery, University Medical Center Groningen, University of Groningen, Groningen, Netherlands; ^3^ Department of Urology, University Medical Center Groningen, University of Groningen, Groningen, Netherlands

**Keywords:** vesicoureteric anastomosis, external ureteric stent, double J stent, urinary tract infection, urological complication

## Abstract

**Introduction:**

Urologic complications (UCs) and urinary tract infections (UTIs) are common after kidney transplantation. Intraoperative stent placement at the vesicoureteric anastomosis reduces UC risk, but increases UTI risk.

**Methods:**

In 2014 our stenting protocol changed from external ureteric stent (ES) to internal double J stent (DJ). We retrospectively studied the occurrence of UCs and UTIs in relation to ES or DJ in 697 kidney recipients.

**Methods:**

An ES was used in 403 patients (57.8%), in 294 (42.2%) a DJ. ES was removed 7-12 days and DJ 3-4 weeks post-operative. Induction immunosuppression was the same in both groups. Primary outcomes at 6 months follow-up were UC (urinary leakage/ureter stenosis) and UTI; they were related to stenting procedure and clinical and transplant characteristics. The incidence of UCs was similar for ES (8.4%) and DJ (6.8%), p=0.389. ES use was a significant risk factor for UTI (OR 1.69 (1.15-2.50), p=0.008). Post-transplant hospitalization was significantly shorter in the DJ group. Despite more acute rejection episodes with ES (ES/DJ: 16.4%/6.1%, p<0.001), no clinical relevant differences in graft outcomes existed.

**Discussion:**

A DJ is, compared to ES, associated with a lower incidence of UTIs and comparable occurrence of UCs and is therefore the preferred technique for stenting the vesicoureteric anastomosis.

## Introduction

For patients with end stage renal disease, kidney transplantation is the preferred treatment option (1). Important risk factors for graft loss and morbidity after kidney transplantation are urological complications (UCs) (2). The most frequent UCs are urinary leakage and ureter stenosis, which are mostly located at the vesicoureteric anastomosis and occur in the first month after kidney transplantation (3). The introduction of the extravesical Lich-Gregoire surgical technique and later on the full thickness surgical technique for the vesicoureteric anastomosis resulted in less UCs (4). In addition, intraoperative placement of a stent at the vesicoureteric anastomosis further contributed to a reduction in UCs (5, 6). Paradoxically, the presence of a stent may introduce other complications, such as urinary tract infections (UTIs) (5, 6), which is the most common infectious complication after kidney transplantion (7, 8). UTIs after kidney transplantation can, like UCs, negatively affect graft- and patient outcomes (9). Currently, there is no consensus about the optimal stenting protocol for preventing UCs and UTIs after kidney transplantation.

Used techniques for stenting the vesicoureteric anastomosis are: an external ureteric stent (ES), which is a flexible catheter that drains externally, percutaneously, from the donor kidney pyelum through the suprapubic bladder region, and the completely internal double J stent (DJ). The use of an ES has several advantages, such as removal without cystoscopy, selective monitoring of the urine output from the kidney graft, and generally a shorter period of a foreign body in situ. However, besides an extra external connection of the urinary tract, hospitalization is generally longer (1-2 weeks post-operative) with ES due to the in-hospital removal of the stent (usually day 7-12 post-transplant) and the indwelling catheter. There is no consensus and very little literature concerning the optimal stent duration for ES usage (10). In contrast, using a DJ, the indwelling catheter usually can be removed in the first postoperative week, and the patient may be discharged. The DJ stent is removed weeks after transplantation *via* cystoscopy, usually in the outpatient setting. There is a large variation in DJ stent duration, usually weeks longer than with ES (11). Overall, as compared to ES, using a DJ contributed to a significant reduction in length of hospital stay and healthcare related costs (12–15). However with the longer duration of the stent, the risk of vesicoureteric reflux and cystoscopic removal may result in more UTIs (6, 11).

Only a few studies comparing the use of a DJ versus (vs.) an ES in kidney transplantation have been performed, using a variety of definitions and presenting varying results regarding incidence of UC and UTI (16–19). In September 2014 our kidney transplantation center changed the intra-operative vesicoureteric anastomosis stenting protocol from using an ES to using a DJ, aiming to reduce post-transplant hospital stay and costs without affecting short- and long term transplant outcomes. The current analysis aimed to clarify whether the change from an ES to a DJ stenting protocol, has indeed resulted in comparable results with respect to UCs, UTIs, patient- and graft outcomes. Furthermore, we aimed to identify possible risk factors for UCs and UTIs in the first months after kidney transplantation.

## Materials and methods

### Patients

We performed a retrospective analysis of all consecutive adult kidney transplant recipients between September 2012 and December 2016 at the University Medical Center Groningen, the Netherlands. Data were obtained using a local registry of all kidney transplant recipients providing the baseline characteristics and kidney transplant outcomes. The data obtained from the patient medical records, contained the type of ureteric stents, indwelling catheter, UCs and UTIs. At admission for kidney transplantation, patients had given consent for using their data for future research and our local Medical Ethical Board waived the need for further consent. All data were anonymized and filed for analyses.

### Transplant procedure

All kidney transplantations were performed by a team of experienced transplant surgeons. The kidney allograft was placed in the right or left iliac fossa *via* an extra-peritoneal approach. The vesicoureteric anastomosis was performed using a modified extravesical Lich-Gregoir technique or a full thickness technique. From September 2012 until September 2014 all kidney transplant recipients received an ES peri-operative. An 8-French, 125 cm enteral feeding tube used as splint, was placed in the graft pyelum and drained externally suprapubic through the bladder wall, hereby stenting the vesicoureteric anastomosis. The ES was removed by gently pulling between 7-12 days post-operative (depending on tightness of the ES and transplant function), without antibiotic prophylaxis. The peri-operatively placed indwelling catheter was usually removed 1-2 days after ES removal and patients were regularly checked for adequate bladder evacuation afterwards. Unto successful indwelling catheter removal, patients stayed in the hospital.

From September 2014 onwards the stenting protocol changed with a transition period of 3 months. In the new protocol a 7-French, 16 cm, internal double J stent was placed peri-operatively, draining from the graft pyelum to the bladder. Based on patient characteristics, like medical history and presence of diuresis, and surgical conditions a ES could still be used. The indwelling catheter was removed on day 5 post-operative and patients were regularly checked for adequate bladder evacuation afterwards. Patients stayed in hospital until successful indwelling catheter removal. The DJ stent was removed 3-4 weeks post-operative at the outpatient clinic *via* cystoscopy. Around cystoscopic DJ stent removal, patients received antibiotic prophylaxis with ciprofloxacin 500mg twice daily, starting 12 hours before unto 24 hours after removal.

The standard immunosuppressive regimen consisted of prednisolone, mycophenolate mofetil and tacrolimus or ciclosporin. All patients received induction immunosuppressive therapy with the IL-2 receptor blocker Basiliximab and perioperative antibiotic prophylaxis with a single dose of cefazoline 2000mg. During the first 6 months after transplantation patients received prophylaxis against *Pneumocystis jiroveci* with co-trimoxazole 400/80 mg once daily.

### Outcomes

Primary outcomes were the occurrence of UCs and/or UTIs during the first six months after kidney transplantation. UCs were defined as urinary leakage or ureter stenosis requiring an intervention (surgical, percutaneous and/or stent placement). Bladder retention, defined as failure of adequate bladder evacuation requiring indwelling catheter placement or intermittent self-catheterization, was not defined as UC because it is not considered a complication related to stenting of the vesicoureteric anastomosis. A UTI was defined as the clinical suspicion of UTI with leukocyturia and/or a positive urinary culture (≥10^5^ CFU/ml) and requiring antibiotic treatment, excluding asymptomatic bacteriuria (ASB). A non-invasive UTI was defined as a UTI without signs or symptoms of tissue invasion and/or bacteremia.

Secondary outcomes were graft function after 6 months [defined as creatinine clearance (ml/min) and proteinuria (g/24h)], and delayed graft function (defined as need for dialysis in the first week after transplantation, with the exception of dialysis to treat immediate post-operative hyperkalemia without further need for kidney replacement therapy during the first week).

### Statistical analysis

Categorical data are presented as number and percentages, continuous data as median and interquartile range (IQR) or mean and standard deviation (depending on data distribution). To compare continuous variables, unpaired t-test or Mann-Whitney U test were used when appropriate. The Chi-square test, Fisher’s exact test or Fisher-Freeman-Halton test were used to compare proportions. Time to occurrence of UTI and UC were analyzed with Kaplan-Meier curves and Log Rank test was used to calculate the Hazard ratio (HR) and the 95% confidence interval (CI) to compare ES and DJ. Univariate analyses were performed to compare groups with and without UTI and UC. Multivariate binary backward and forward LR logistic regression was performed with the use of ES or DJ and additional variables with a p-value ≤0.1 by univariate analysis. Thereafter the binary logistic regression was repeated with method enter with the remaining significant variables and the use of ES or DJ to identify the possible influence of the used type of stent on the occurrence of UTI and UC. Multiple imputation (5 times) was performed for missing values and binary logistic regression was repeated with the imputed data to verify that the missing values did not influence the outcome. All reported p-values are two tailed and a p-value <0.05 was considered statistically significant. Analyses were conducted with SPSS version 23 (IBM corp., Armonk, USA) and GraphPad Prism version 9 (GraphPad Software, La Jolla, USA).

## Results

### Patient characteristics

During the inclusion period, 711 consecutive kidney transplants were performed of which 14 were excluded due to graft loss ≤1 week after transplantation. Of the remaining 697 kidney transplants, 13 were lost to follow-up for UC as outcome (deceased n=7, transplantectomy n=4, transplant failure without transplantectomy n=2) and 10 were lost to follow-up for UTI as outcome (deceased n=4, transplantectomy n=4, transplant failure without transplantectomy n=2). In 403 (57.8%) patients an ES was placed during the transplant procedure and in 294 (42.2%) patients a DJ. After December 2014, in 107 cases a ES was used due to patient and/or surgical characteristics. The mean recipient age at transplantation was 53 years for both groups and 40% were women. Patient characteristics of the ES and DJ group were mostly similar (**Table 1**). Pre-transplant diuresis volume was higher in the ES group (ES 1500 (IQR 187.5-2000) ml/24 hours, DJ 500 (IQR 0-1925) ml/24 hours; p<0.001) as was the number of pre-emptive transplantation (ES 33.3% vs. DJ 22.4%). There was no difference in donor type or presence of pre-transplant urologic history (ES 47.6% vs. DJ 52.4%; p=0.217).

**Table 1:** Patient characteristics.

Variable	External ureteric stent n=403	Double J stent n=294	p-value
Age at presentation (years)	52.5 ± 13.5	52.7 ± 13.8	0.828
Gender (men/women)	240 (59.6)/163 (40.4)	174 (59.2)/120 (40.8)	0.922
BMI at transplantation (kg/m^2^)	26.3 ± 4.5	26.1 ± 4.4	0.694
Smoking	83 (20.6)	55 (18.7)	0.537
Original urological condition	110 (27.3)	69 (23.5)	0.254
Prior transplantation	36 (8.9)	60 (20.4)	<0.001*
Anuric pre-transplant (n=687)	77 (19.6)	91 (31.0)	0.001*
Pre-transplant diuresis (ml/24 hours) (n=687)	1500.0 (187.5-2000.0)	500.0 (0.0-1925.0)	<0.001*
Renal replacement therapy			0.003*
- None (pre-emptive transplantation)	137 (34.0)	66 (22.4)	
- Hemodialysis	187 (46.4)	167 (56.8)	
- Peritoneal dialysis	79 (19.6)	61 (20.7)	
Time on dialysis (months) (n=488)	29.0 (17.0-49.5)	27.0 (16.0-45.0)	0.421
Type of donor			0.565
- Donation after Brain Death	100 (24.8)	66 (22.4)	
- Donation after Circulatory Death	83 (20.6)	73 (24.8)	
- Living unrelated	136 (33.7)	99 (33.7)	
- Living related	84 (20.8)	56 (19.0)	
ABO-incompatible transplantation	6 (1.5)	7 (2.4)	0.390
HLA mismatching	3.1 ± 1.6	3.3 ± 1.5	0.301
***Urologic history^a^***	192 (47.6)	154 (52.4)	0.217
History of UTI	94 (23.3)	78 (26.5)	0.332
Vesicoureteral reflux	11 (2.7)	9 (3.1)	0.796
Hydronephrosis	6 (1.5)	8 (2.7)	0.283
Cystic kidney disease	81 (20.1)	47 (16.0)	0.166
Nephrolithiasis/urolithiasis	22 (5.5)	15 (5.1)	0.836
Urethral stricture	7 (1.7)	3 (1.0)	0.513
Ureteral stenosis	3 (0.7)	2 (0.7)	1.000
Malignancy urinary tract	9 (2.2)	16 (5.4)	0.024*
- Prostate cancer	3 (0.7)	4 (1.4)	
- Urothelial carcinoma	2 (0.5)	3 (1.0)	
- Renal cell carcinoma	3 (0.7)	8 (2.7)	
- Other	1 (0.2)	1 (0.3)	
Urostomy (Bricker conduit)	3 (0.7)	2 (0.7)	1.000
Presence of urinary catheter	11 (2.7)	5 (1.7)	0.370
- Indwelling catheter	0 (0.0)	1 (0.3)	
- Suprapubic catheter	0 (0.0)	0 (0.0)	
- Double-J	1 (0.2)	0 (0.0)	
- Nephrostomy	0 (0.0)	2 (0.7)	
- Clean intermittent self-catheterization	10 (2.5)	2 (0.7)	
Urinary tract surgery	62 (15.4)	79 (26.9)	<0.001*
Bladder dysfunction	8 (2.0)	3 (1.0)	0.372
Intraoperative parameters			
Cold ischemia time (minutes)	204 (152-776)	224 (155-724)	0.965
First warm ischemia time (minutes) (n=694)	3 (2-6)	3 (2-9)	0.943
Second warm ischemia time (minutes) (n=696)	41 (35-48)	38 (32-45)	<0.001*

Categorical data reported as number (%); continuous data reported as median (interquartile range), or as mean ( ± standard deviation), depending on data distribution. BMI, body mass index; HLA, Human leukocyte antigen. ^a^Cases could be part of more than one group. *P-value <0.05, statistical significant.

A reduction in hospital stay after kidney transplantation was seen after the change in protocol, median of 7 (IQR 9-14) days after DJ placement and a median of 15 (IQR 13-17) days after ES placement (p<0.001). As expected, duration of stent placement was longer in the DJ group compared to the ES group (31.5 days vs. 12 days; p<0.001). With respect to clinical outcome (6 months follow-up), no clear differences were found in the occurrence of bladder retention (ES 3.5% vs. DJ 3.1%; p=0.767) and serum creatinine (**Table 2**). Calculated creatinine clearance showed a minor but clinical not relevant difference between ES and DJ (61.5 ml/min vs. 66.6 ml/min). Acute rejection was seen more frequently in the ES group (16.4%) compared to the DJ group (6.1%).

**Table 2:** Posttransplant results.

Variable	External ureteric stent n=403	Double J stent n=294	p-value
Duration of hospital stay (days)	15 (13-17)	7 (9-14)	<0.001*
Stent duration after transplantation (days) (n=679)	12 (7-12)	31.5 (27-38)	<0.001*
Indwelling catheter duration after transplantation (days) (n=659)	14 (9-14)	6 (5-7.8)	<0.001*
Bladder catheter replacement within 48 hours after removal	16 (4.0)	14 (4.8)	0.611
- Indwelling catheter	16 (4.0)	12 (4.1)	
- Clean intermittent self-catheterization	0 (0.0)	2 (0.7)	
Delayed graft function^a^	88 (21.8)	60 (20.4)	0.649
Bladder retention/non-adequate bladder evacuation^b^	14 (3.5)	9 (3.1)	0.767
Acute rejection^c^	66 (16.4)	18 (6.1)	<0.001*
- Early rejection/late rejection	46 (69.7)/20 (30.3)	15 (83.3)/3 (16.7)	0.373
Oliguria after transplantation	101 (25.1)	69 (23.5)	0.629
- Duration of oliguria (days)	9.0 (3.5-15.0)	7.0 (3.0-10.5)	0.126
HD after transplantation	82 (20.3)	55 (18.7)	0.591
- Frequency of HD after transplantation	4.5 (2.0-8.0)	3.0 (2.0-5.0)	0.019*
CAPD after transplantation	6 (1.5)	5 (1.7)	1.000
- Days of CAPD after transplantation	7.0 (3.3-10.0)	6.0 (3.0-8.5)	0.641
Serum creatinine level (µmol/L)			
- First visit outpatient clinic after transplantation (n=683)	139 (111.0-190.0)	146 (110.0-191.0)	0.996
- 6 months after transplantation (n=674)	130 (107.0-161.0)	131 (109.0-163.0)	0.937
Creatinine clearance (ml/min)			
- First visit outpatient clinic after transplantation(n=583)	54.1 ± 22.8	57.2 ± 22.8	0.108
- 6 months after transplantation (n=580)	61.5 ± 23.6	66.6 ± 24.9	0.012*
Proteinuria during follow-up^d^			
- First visit outpatient clinic after transplantation (n=630)	225 (59.4)	158 (62.9)	0.367
- Level of proteinuria (g/24h)	0.5 (0.3-0.8)	0.5 (0.3-0.7)	0.983
- 6 months after transplantation (n=575)	139 (40.5)	57 (24.6)	<0.001*
- Level of proteinuria (g/24h)	0.4 (0.3-0.7)	0.4 (0.3-0.8)	0.374

Categorical data reported as number (%); continuous data reported as median (interquartile range), or as mean ( ± standard deviation), depending on data type. CAPD, Continuous Ambulant Peritoneal Dialysis; HD, Hemodialysis.

^a^Defined as need of dialysis in the first week after transplantation and/or transplantectomy within the first week after transplantation. ^b^Defined as failure of adequate bladder evacuation requiring indwelling catheter placement or start of clean intermittent self-catheterization.^c^Defined as need for anti-rejection therapy (methylprednisolone, anti-thymocyte globulin and/or alemtuzumab). Early rejection: rejection ≤30 days after transplantation, late rejection: rejection >30 days and ≤6 months after transplantation. ^d^Defined as ≥0.3 g/24uur. *P-value <0.05, statistical significant.

### Urological complications

**Figure 1:**
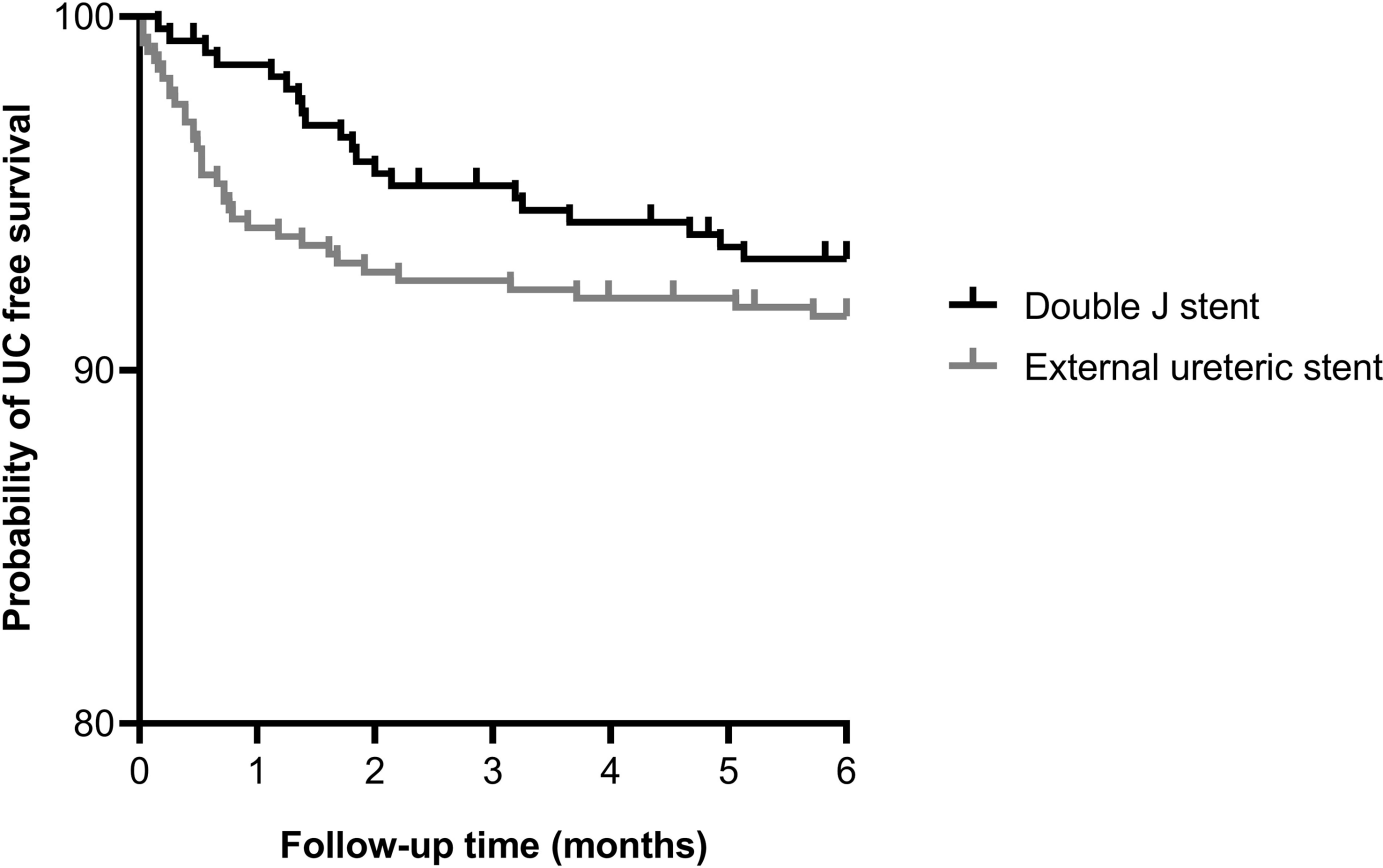
Kaplan-Meier curve showing the probability of urological complication free survival within 6 months after kidney transplantation for external ureteric stent (n=403, UC n=34) and double J stent (n=294, UC n=20); HR 1.27 (95% CI 0.74-2.18), p=0.389. Thirteen cases were censored before an urological complication occurred.

The occurrence of an UC requiring intervention within 6 months after transplantation was similar between the ES group and DJ group; 34 (8.4%) vs. 20 (6.8%) patients, p=0.389, HR 1.27 (95% CI 0.74-2.18) (**Figure 1**). Also, no differences existed between the incidence of urinary leakage and ureter stenosis (**Table 3**). In both the ES and DJ group, the majority of the UCs occurred in the first month after transplantation, but there still was a significant difference in UC occurrence in favor of DJ (ES 24 (6.0%) vs. DJ 4 (1.4%) patients, p=0.002, HR 4.48 (95% CI 2.12-9.48) (**Supplemental Figure 1**). After one month of follow-up there was a clear lower incidence of urinary leakage and ureter stenosis in the DJ group. In addition, almost all urinary leakages occurred in the first month after transplantation (**Table 3**).

**Table 3:** Urological complications requiring intervention.

Variable	External ureteric stent n=403	Double J stent n=294	p-value
One month of follow-up			
Type of urological complication			
- Urinary leakage	12 (3.0)	2 (0.7)	0.033*
- Ureter stenosis	17 (4.2)	2 (0.7)	0.005*
Six months of follow-up			
Type of urological complication			
- Urinary leakage	12 (3.0)	3 (1.0)	0.079
- Ureter stenosis	27 (6.7)	17 (5.8)	0.623

Categorical data reported as number (%). In 5 cases both urinary leakage and ureter stenosis occured. *P-value <0.05, statistical significant.

In univariate analysis, multiple risk factors for developing an UC within 6 months after kidney transplantation were identified (**Table 4**). These factors mainly relate to kidney donor quality and posttransplant urinary flow. In both groups no relation existed between duration (days) of stent placement and the occurrence of UC. In 5 kidney transplant procedures both an acute rejection and a UC occurred, in 4 (80%) the acute rejection occurred prior to the UC. In 1 (20%) case the acute rejection was detected after the UC and if this case was defined as no acute rejection the difference between those with and without UC in acute rejection remains unchanged. Both backward and forward logistic regression analysis, with the occurrence of UC as dependent outcome, failed to show the independent contribution of ≥1 variable, whether ES/DJ use was factored in or not. The use of imputed data did not change these results.

**Table 4:** Analysis of risk factors for an urologic complication within 6 months after kidney transplantation.

Variable		No urologic complication n=643		Urologic complication n=54	p-value
Age at presentation (years)		52.4 ± 13.5		54.4 ± 15.5	0.306
Gender (men/women)		377 (58.6)/266 (41.4)		37 (68.5)/17 (31.5)	0.155
BMI at transplantation (kg/m^2^)		26.2± 4.5		26.2± 4.1	0.940
Smoking		521 (81.0)		38 (70.4)	0.059
Original urological condition		166 (25.8)		13 (24.1)	0.778
Prior transplantation		88 (13.7)		8 (14.8)	0.817
Anuric pretransplant (n=687)		153 (24.2)		15 (27.8)	0.554
Pre-transplant diuresis (ml/24 hours) (n=687)		1000.0 (100-2000.0)		500.0 (0.0-1750.0)	0.084
Renal replacement therapy					0.199
- None (pre-emptive transplantation)		193 (30.0)		10 (18.5)	
- Hemodialysis		323 (50.2)		31 (57.4)	
- Peritoneal dialysis		127 (19.8)		13 (24.1)	
Time on dialysis (months) (n=488)		28.0 (16.0-46.0)		33.0 (20.0-50.0)	0.391
Type of donor^a^					0.104
- Donation after Brain Death		146 (22.7)		20 (37.0)	
- Donation after Circulatory Death		144 (22.4)		12 (22.2)	
- Living unrelated		221 (34.4)		14 (25.9)	
- Living related		132 (20.5)		8 (14.8)	
ABO-incompatible transplantation		12 (1.9)		1 (1.9)	1.000
HLA mismatching		3.2 ± 1.5		3.4 ± 1.6	0.337
Urologic history		324 (50.4)		22 (40.7)	0.173
- History of UTI		164 (25.2)		10 (18.5)	0.274
Cold ischemia time, min (n=696)		203.5 (153.0-740.3)		614.0 (170.3-800.0)	0.034*
First warm ischemia time, min (n=694)		3.0 (2.0-6.0)		3.0 (0-7.8)	0.545
Second warm ischemia time, min (n=696)		39.0 (33.0-46.0)		43.5 (35.0-4.3)	0.064
Duration of hospital stay (days)		14 (9-16)		17 (13.8-24.3)	<0.001*
External ureteric stent/Double J stent		369 (57.4)/274 (42.6)		34 (63.0)/20 (37.0)	0.426
Stent duration after transplantation (days)					
- External ureteric stent (n=387)		12.0 (7.0-12.0)		12.0 (8.0-12.0)	0.340
- Double J stent (n=292)		31.0 (27.0-38.0)		35.0 (26.5-40.0)	0.457
Indwelling catheter duration after transplantation (days)					
- External ureteric stent (n=375)		14.0 (9.0-14.0)		14.0 (14.0-17.5)	<0.001*
- Double J stent (n=284)		6.0 (5.0-7.0)		6.0 (5.5-10.0)	0.052
Bladder catheter replacement within 48 hours after removal		27 (4.2)		3 (5.6)	0.500
Delayed graft function^a^		129 (20.1)		19 (35.2)	0.009*
Bladder retention/non-adequate bladder evacuation^b^		22 (3.4)		1 (1.9)	1.000
Acute rejection^c^		79 (12.3)		5 (9.3)	0.512
- Early rejection/late rejection		526 (70.9)/23 (29.1)		5 (100.0)/0 (0.0)	0.316
Oliguria after transplantation		148 (23.0)		22 (40.7)	0.004*
- Duration of oliguria (days)		8.0 (3.0-12.0)		11.0 (3.5-17.5)	0.231
HD after transplantation		118 (18.4)		19 (35.2)	0.003*
- Frequency of HD after transplantation		3.0 (2.0-6.0)		4.0 (1.0-9.0)	0.739
CAPD after transplantation		11 (1.7)		0 (0.0)	1.000
- Days of CAPD after transplantation		7.0 (4.0-10.0)		NA	NA
Serum creatinine level (µmol/L)					
- First visit outpatient clinic after transplantation (n=683)		138 (110.0-187.0)		170 (139.5-249.0)	<0.001*
- 6 months after transplantation(n=674)		129 (107.0-160.0)		158 (134.0-207.8)	<0.001*
Creatinine clearance (ml/min)					
- First visit outpatient clinic after transplantation (n=583)		56.3 ± 22.8		43.6 ± 19.4	0.001*
- 6 months after transplantation (n=580)		64.7 ± 24.0		49.3 ± 23.2	<0.001*
Proteinuria during follow-up^d^					
- First visit outpatient clinic after transplantation (n=630)		346 (59.5)		37 (77.1)	0.016*
- Level of proteinuria (g/24h)		0.5 (0.3-0.7)		0.7 (0.5-1.1)	0.001*
- 6 months after transplantation (n=575)		170 (32.0)		26 (60.5)	<0.001*
- Level of proteinuria (g/24h)		0.4 (0.3-0.6)		0.5 (0.3-1.0)	0.163

Categorical data reported as number (%); continuous data reported as median (interquartile range), or as mean ( ± standard deviation), depending on data distribution. BMI, body mass index; CAPD, Continuous Ambulant Peritoneal Dialysis; HD, Hemodialysis; HLA, Human leukocyte antigen; NA, not applicable; UTI, Urinary tract infection. ^a^Defined as need of dialysis in the first week after transplantation and/or transplantectomy within the first week after transplantation. ^b^Defined as failure of adequate bladder evacuation requiring indwelling catheter placement or start of clean intermittent self-catheterization.^c^Defined as need for anti-rejection therapy (methylprednisolone, anti-thymocyte globulin and/or alemtuzumab). Early rejection: rejection ≤30 days after transplantation, late rejection: rejection >30 days and ≤6 months after transplantation. ^d^Defined as ≥0.3 g/24uur.*P-value <0.05, statistical significant.

### Urinary tract infections

**Figure 2:**
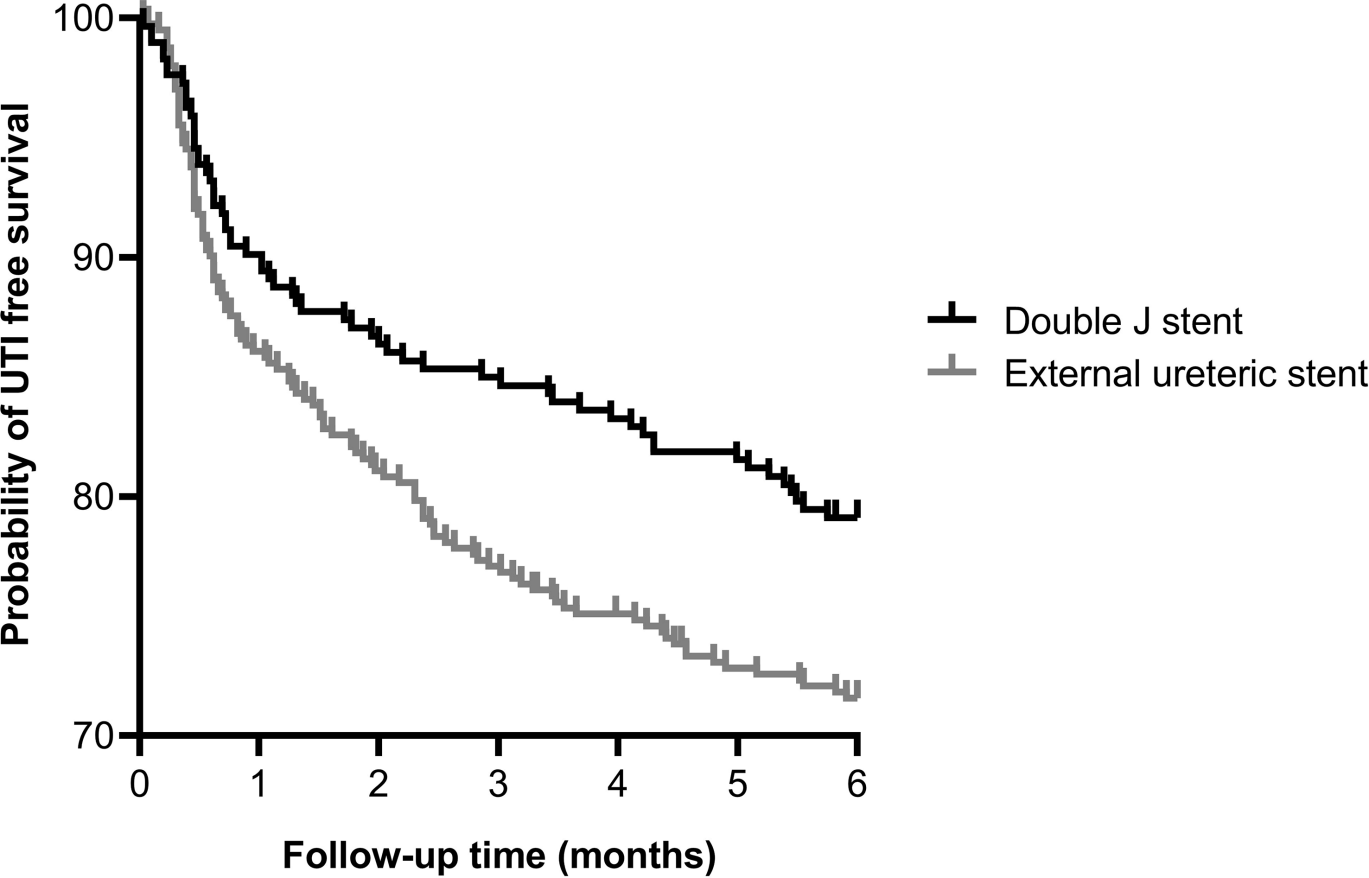
Kaplan-Meier curve showing the probability of UTI free survival within 6 months after kidney transplantation for external ureteric stent (n=403, UTI n=114) and double J stent (n=294, UTI n=61); HR 1.43 (95% CI 1.06–1.93), p=0.023. Ten cases were censored before a urinary tract infection occurred.

In the ES group more patients experienced at least one UTI within the first 6 months after kidney transplantation compared to the DJ group: 114 (28.3%) vs. 61 (20.7%) patients, p=0.023, HR 1.43 (95% CI 1.06-1.93) (**Figure 2**). Both non-invasive UTIs and invasive UTIs were more common in the ES group. The occurrence of multiple UTIs within 6 months of follow-up did not differ between ES and DJ (**Table 5**). No differences in urine diagnostic test results of the first UTI episode between ES and DJ existed; in both groups UTI was urine culture proven in around 86% (data not shown). The number of UTIs per month (expressed as total amount of UTI’s per person divided by the duration of the follow-up period) did not differ between ES and DJ (0.08 UTI/month vs. 0.07 UTI/month). In the UTI group, in 13.8% the first UTI occurred before the ES was removed, while in the DJ group in 60.0% the first UTI occurred before the DJ was removed. A UTI during post-transplantation hospitalization was more common in the ES group, probably influenced by longer hospitalization and indwelling catheter placement as per protocol (ES 10.9% vs. DJ 6.1%, p=0.028). At one month of follow-up, the first UTI occurred in 85 cases with a comparable incidence between ES and DJ; 56 (13.9%) vs. 29 (9.9%), p=0.109, HR 1.44 (95% CI 0.94-2.21) (**Supplemental Figure 2**). Between one and six months of follow-up, the first UTI occurred in 90 cases (ES n=58 and DJ n=32).

**Table 5:** Urinary tract infections within 6 months of follow-up.

Variable	External ureteric stent n=403	Double J stent n=294	p-value
Type of first UTI within 6 months of follow-up			0.047*
- No UTI	289 (71.7)	233 (79.3)	
- Non-invasive UTI	85 (21.1)	44 (15.0)	
- Pyelonephritis	2 (0.5)	4 (1.4)	
- Urosepsis	27 (6.7)	13 (4.4)	
First UTI during post transplantation hospitalization	44 (10.9)	18 (6.1)	0.028*
Hospital admission due to first UTI	26 (6.5)	17 (5.8)	0.717
Multiple UTIs within 6 months of follow-up	44 (10.9)	30 (10.2)	0.762

Categorical data reported as number (%). *P-value <0.05, statistical significant.

In univariate analysis, multiple factors associated with the development of a UTI within 6 months after kidney transplantation were identified (**Table 6**). In both the ES and DJ group no relation was found between duration (days) of stent placement and the occurrence of a UTI. In 34 kidney transplant procedures both an acute rejection and a UTI occurred. In 27 (79.4% the acute rejection occurred prior to the first UTI and in 7 (20.6%) the acute rejection was detected after the first UTI. If these 7 cases are defined as no acute rejection the difference between those with and without UTI in acute rejection remains significant. Urological complications were more common in the UTI group compared to the no UTI group (17.1% vs. 4.6%, p<0.001). In 30 kidney transplant procedures both a UC and a UTI occurred within 6 months after transplantation. In 21 (70%) cases the UC was discovered prior to the first UTI and in 9 cases the UC was detected simultaneously with or shortly after a UTI. If these 9 cases are defined as no UC the difference between those with and without UTI in UC remains significant. In the final multivariate logistic regression model the type of stenting procedure, ES or DJ, remained significantly associated with the occurrence of an UTI (OR 1.69 (1.15-2.50), p=0.008). Other factors were age, gender, history of UTI, indwelling catheter replacement within 48 hours after removal, and occurrence of urological complications during follow-up (**Table 7**). The use of imputed data did not change these results.

**Table 6:** Analysis of risk factors for a urinary tract infection within 6 months after kidney transplantation.

Variable	No UTI n=522	UTI n=175	p-value
Age at presentation (years)	51.5 ± 13.8	55.6 ± 12.8	<0.001*
Gender (men/women)	333 (63.8)/189 (36.2)	81 (46.3)/94 (53.7)	<0.001*
BMI at transplantation (kg/m^2^)	26.1 ± 4.5	26.5 ± 4.5	0.389
Smoking	108 (20.7)	30 (17.1)	0.308
Original urological condition	125 (23.9)	54 (30.9)	0.070
Prior transplantation	69 (13.2)	27 (15.4)	0.463
Anuric pretransplant (n=687)	123 (23.9)	45 (26.2)	0.547
Pre-transplant diuresis (ml/24 hours) (n=687)	1200 (100-2000)	850 (0-2000)	0.054
Renal replacement therapy			0.371
- None (pre-emptive transplantation)	159 (30.5)	44 (25.1)	
- Hemodialysis	262 (50.2)	92 (52.6)	
- Peritoneal dialysis	101 (19.3)	39 (22.3)	
Time on dialysis (months) (n=488)	27.0 (15.0-44.0)	34.5 (18.0-51.0)	0.048*
Type of donor			0.011*
- Donation after Brain Death	110 (21.1)	56 (32.0)	
- Donation after Circulatory Death	116 (22.2)	40 (22.9)	
- Living unrelated	181 (34.7)	54 (30.9)	
- Living related	115 (22.0)	25 (14.3)	
ABO-incompatible transplantation	10 (1.9)	3 (1.7)	1.000
HLA mismatching	3.1 ± 1.5	3.3 ± 1.5	0.213
Urologic history	234 (44.8)	112 (64.0)	<0.001*
- History of UTI	100 (19.2)	72 (41.1)	<0.001*
Cold ischemia time, min (n=696)	192.0 (152.0-726.5)	510.0 (160.3-803.0)	0.011*
First warm ischemia time, min (n=694)	3 (2-6)	3 (0-7)	0.220
Second warm ischemia time, min (n=696)	39 (33-46)	40 (34-47)	0.460
Duration of hospital stay (days)	13 (8-15)	15 (10-22)	<0.001*
External ureteric stent/Double J stent	289 (55.4)/233 (44.6)	114 (65.1)/61 (34.9)	0.023*
Stent duration after transplantation (days)			
- External ureteric stent (n=387)	12 (7-12)	12 (7.5-12)	0.584
- Double J stent (n=292)	31 (27-38)	33 (25-40.5)	0.823
Indwelling catheter duration after transplantation (days)			
- External ureteric stent (n=375)	14 (9-14)	14 (13-14.3)	0.013*
- Double J stent (n=284)	6 (5-7)	6 (5-8)	0.068
Bladder catheter replacement within 48 hours after removal	10 (1.9)	20 (11.4)	<0.001*
Delayed graft function^a^	104 (19.9)	44 (25.1)	0.144
Bladder retention/non-adequate bladder evacuation^b^	9 (1.7)	14 (8.0)	<0.001*
Acute rejection^c^	50 (9.6)	34 (19.4)	0.001*
- Early rejection/late rejection	39 (78.0)/11 (22.0)	22 (64.7)/12 (35.3)	0.180
Oliguria after transplantation	121 (23.2)	49 (28.0)	0.199
- Duration of oliguria (days)	7.0 (2.5 -12.0)	11.0 (7.0-17.5)	0.003*
HD after transplantation	97 (18.6)	40 (22.9)	0.218
- Frequency of HD after transplantation	3 (2-6)	4.5 (3-8.8)	0.010*
CAPD after transplantation	7 (1.3)	4 (2.3)	0.481
- Days of CAPD after transplantation	6.0 (2.0-10.0)	7.0 (4.8-9.3)	0.499
Serum creatinine level (µmol/L)			
- First visit outpatient clinic after transplantation (n=683)	141 (113.0-185.8)	140 (105.0-210.0)	0.767
- 6 months after transplantation(n=674)	131 (108.3-161.0)	127 (104.0-164.3)	0.585
Creatinine clearance (ml/min)			
- First visit outpatient clinic after transplantation(n=583)	57.8 ± 22.6	48.0 ± 22.0	<0.001*
- 6 months after transplantation (n=580)	65.9 ± 23.8	56.2 ± 24.3	<0.001*
Proteinuria during follow-up^d^			
- First visit outpatient clinic after transplantation (n=630)	283 (60.1)	100 (62.9)	0.531
- Level of proteinuria (g/24h)	0.5 (0.3-0.7)	0.5 (0.3-0.8)	0.439
- 6 months after transplantation (n=575)	135 (30.8)	61 (44.5)	0.003*
- Level of proteinuria (g/24h)	0.4 (0.3-0.7)	0.4 (0.3-0.7)	0.586
Urological complication within 6 months of follow-up^e^	24 (4.6)	30 (17.1)	<0.001*
- Urinary leakage	7 (1.3)	8 (4.6)	
- Ureter stenosis	19 (3.6)	25 (14.3)	

Categorical data reported as number (%); continuous data reported as median (interquartile range), or as mean ( ± standard deviation), depending on data distribution. BMI, body mass index; CAPD, Continuous Ambulant Peritoneal Dialysis; HD, Hemodialysis; HLA, Human leukocyte antigen; UTI, Urinary tract infection. ^a^Defined as need of dialysis in the first week after transplantation and/or transplantectomy within the first week after transplantation. ^b^Defined as failure of adequate bladder evacuation requiring indwelling catheter placement or start of clean intermittent self-catheterization.^c^Defined as need for anti-rejection therapy (methylprednisolone, anti-thymocyte globulin and/or alemtuzumab). Early rejection: rejection ≤30 days after transplantation, late rejection: rejection >30 days and ≤6 months after transplantation. ^d^Defined as ≥0.3 g/24uur. ^e^In 5 cases both urinary leakage and ureter stenosis occured. *P-value <0.05, statistical significant.

**Table 7:** Multivariate analysis of factors associated with a urinary tract infection within 6 months after transplantation.

Variable	Odds ratio (95% CI)	p-value
Age at presentation (years)	1.03 (1.01-1.04)	0.001*
Gender (men/women)	0.46 (0.31-0.68)	<0.001*
History of UTI	3.00 (1.99-4.54)	<0.001*
External ureteric stent/Double J stent	1.69 (1.15-2.50)	0.008*
Bladder catheter replacement within 48 hours after removal	7.59 (3.29-17.52)	<0.001*
Urological complications	5.67 (3.05-10.51)	<0.001*

n=697. Nagelkerke R^2^ 0.227, constant OR 0.043, p-value <0.001 *P-value <0.05, statistical significant.

## Discussion

Prophylactic stenting of the vesicoureteric anastomosis in kidney transplantation is nowadays standard practice in most kidney transplant centers. There is no consensus on the optimal stenting type and duration (10, 11, 16–19). We compared the outcomes on UC and UTI incidence between a DJ and ES stenting protocol.

Our reported UC rates are 8.5% in the ES group vs. 6.8% in the DJ group. Previous research has shown comparable incidences ranging from 0.3-25.0% for ES vs. 0.0-5.4% for DJ (16–19). For UTI incidence, the reported rates in the literature are ranging from 12.5-41.4% for ES vs. 7.6-51.5% for DJ (16–18). Our cohort showed an UTI rate of 28.4% in the ES group vs. 20.9% in the DJ group, both are in the range of previous studies. Compared to the literature, all patients in our analysis were transplanted using the same immunosuppressive regimen, including Basiliximab induction. All patients received co-trimoxazole prophylaxis during the first 6 months after transplantation, whether this contributed to our UTI incidence is questionable. In the only randomized study performed, standard co-trimoxazole prophylaxis had no significant influence on UTI occurrence post-transplantation (20).

The ES group showed significant more UCs in the first month after transplantation compared to the DJ group. After the first month, there is a comparable incidence of UC over the follow-up time. Earlier research also showed that the majority of the UCs occur in the first 4 weeks after transplantation (21). Shorter stent duration in the ES group, 12 days vs. 31.5 days in DJ, could have led to increased early UCs. But, this will not have influenced our results, because each group within our analysis showed no association of stent duration and UC, nor was any other clinical relevant risk factor for UC identified.

Patients in the ES group experienced more UTIs, especially during the first month postoperative, which may be due to the longer presence of the indwelling catheter compared to DJ use and an extra exterior connection of the urinary tract through the percutaneous position of the ES. This is in line with literature, stating that the duration an indwelling catheter is *in situ* is a risk factor for the development of bacteriuria, especially starting 1-2 weeks after placement (22, 23). Multivariate analysis showed that using an ES and UC are significant and independent risk factors for the occurrence of UTI. Since our data showed more UCs in the ES group, especially in the first month, this could also explain the higher UTI rate in this group. Patients in the DJ group more often had an urological history prior to transplantation, but this was not identified as a risk factor for UTIs. A pre-transplant history of UTIs proved to be a risk factor for post-transplantation UTI. Although there were slightly more patients with a history of UTI in the DJ group, they showed better outcomes on UTIs compared to ES. Likewise older age and female gender were associated with a UTI, but these factors did not influence the difference in UTI between the ES and DJ, since there were no baseline differences between both groups.

In our cohort DJ stent removal was performed after a median of 31.5 days (27–38), which is longer compared to earlier studies (11). The longer duration could potentially have led to more UTIs (24). Although around 60% of the first UTIs in the DJ group occurred before stent removal, our analysis failed to show a relation between stent duration and the occurrence of UTI. In addition, the incidence of UTI was lower in the DJ group as compared to the ES group, despite the fact that the stent was a substantially shorter period *in situ* in the latter group. Almost all our DJ stent removal procedures were done with antibiotic prophylaxis whereas ES removal is done without, which could have led to more UTIs in the ES group. The prophylaxis only consisted of a short course of antibiotics; therefore we assume there is no or minor influence on UTI incidence.

Although patients with an ES experienced (slightly) more UCs and UTIs, there were no clinical relevant differences in graft outcome 6 months after kidney transplantation. Creatinine clearance was slightly, but significantly, lower in the ES group and in these patients proteinuria was more often present. The higher rates of UCs and UTIs in the ES group could have led to these small, but clinically irrelevant differences (25, 26). Interestingly, there was a significant higher incidence of acute allograft rejection in the ES group, most often in the first 30 days after transplantation, which may also have led to the small differences in graft outcomes at 6 months. We were unable to identify a clear cause for this difference in rejection episodes, as baseline immunological characteristics were comparable between the ES and DJ group. It has been suggested that UCs and UTIs may have a role in triggering immunological phenomena like allograft rejection (27, 28) and vice versa, acute rejection is also a risk factor for early UCs (20). Our analysis showed no association between acute rejection and incidence of UC or UTI. The unexplained increase in acute rejection episodes when using an ES has been reported by others and may also have something to do with more selective monitoring of graft urinary output leading to more biopsies in the early post-operative phase (16, 19, 29).

This study has a few strengths and limitations that need to be addressed. A major strength is the comparable circumstances and baseline characteristics between the two groups. Second, we described two large, representative groups with a longer follow-up compared to previous published cohorts. A limitation of our study is the retrospective design. Although we reviewed all the patients charts and used our local kidney transplantation database, it could be that the UCs and UTIs are not registered correctly. In contrast to other studies, we excluded patients with ASB as having a UTI since there is no need for treatment. This could have led to better results on UTI occurrence, but should not have influenced patient- and graft outcomes. A recent systematic review showed that after kidney transplantation untreated ASB vs. treated ASB have the same outcomes regarding graft function and risk of getting a symptomatic UTI (30). Lastly, because we used data from several years, it could be that modernization of healthcare techniques and improved quality of care has led to better results for the DJ group.

Overall, using a DJ, compared to an ES, for stenting the vesicoureteric anastomosis after kidney transplantation, leads to significantly less urinary tract infections and a shorter hospitalization period post-transplantation which reduces healthcare related costs. Incidence of urological complications was comparable for both groups. There is no indication that a DJ performs worse compared to an ES with regards to graft outcomes. Our data suggests that use of a DJ is the preferred technique for stenting the vesicoureteric anastomosis during kidney transplantation.

## Data availability statement

The raw data supporting the conclusions of this article will be made available by the authors, without undue reservation.

## Ethics statement

Ethical review and approval was not required for the study on human participants in accordance with the local legislation and institutional requirements. Written informed consent for participation was not required for this study in accordance with the national legislation and the institutional requirements.

## Author contributions

IH participated in data collection, database editing, data analysis and manuscript writing. SM participated in research design, performance of research, data collection, database editing, data analysis, and manuscript writing. AJ, RP, and J-SS participated in research design and manuscript editing. JR participated in data collection and manuscript editing. BD participated in manuscript editing. CS participated in research design, data collection, database editing, data analysis and manuscript editing. IH and SM contributed equally as first author. All authors contributed to the article and approved the submitted version.

## Supplementary material

Supplementary Figure 1 Kaplan-Meier curve showing the probability of urological complication free survival within 1 month after kidney transplantation for external ureteric stent (n=403, UC n=24) and double J stent (n=294, UC n=4); HR 4.48 (95% CI 2.12-9.48), p=0.002. Four cases were censored before an urological complication occurred.

Supplementary Figure 2 Kaplan-Meier curve showing the probability of UTI free survival within 1 month after kidney transplantation for external ureteric stent (n=403, UTI n=56) and double J stent (n=294, UTI n=29); HR 1.44 (95% CI 0.94-2.21), p=0.109. Three cases were censored before a urinary tract infection occurred.
