# Supplementary figures and images for: External ureteric stent versus internal double J stent in kidney transplantation: a retrospective analysis on the incidence of urological complications and urinary tract infections
Source: Front Nephrol. 2023 May 16;3:1130672. doi: 10.3389/fneph.2023.1130672 (PMC10479681; doi:10.3389/fneph.2023.1130672)

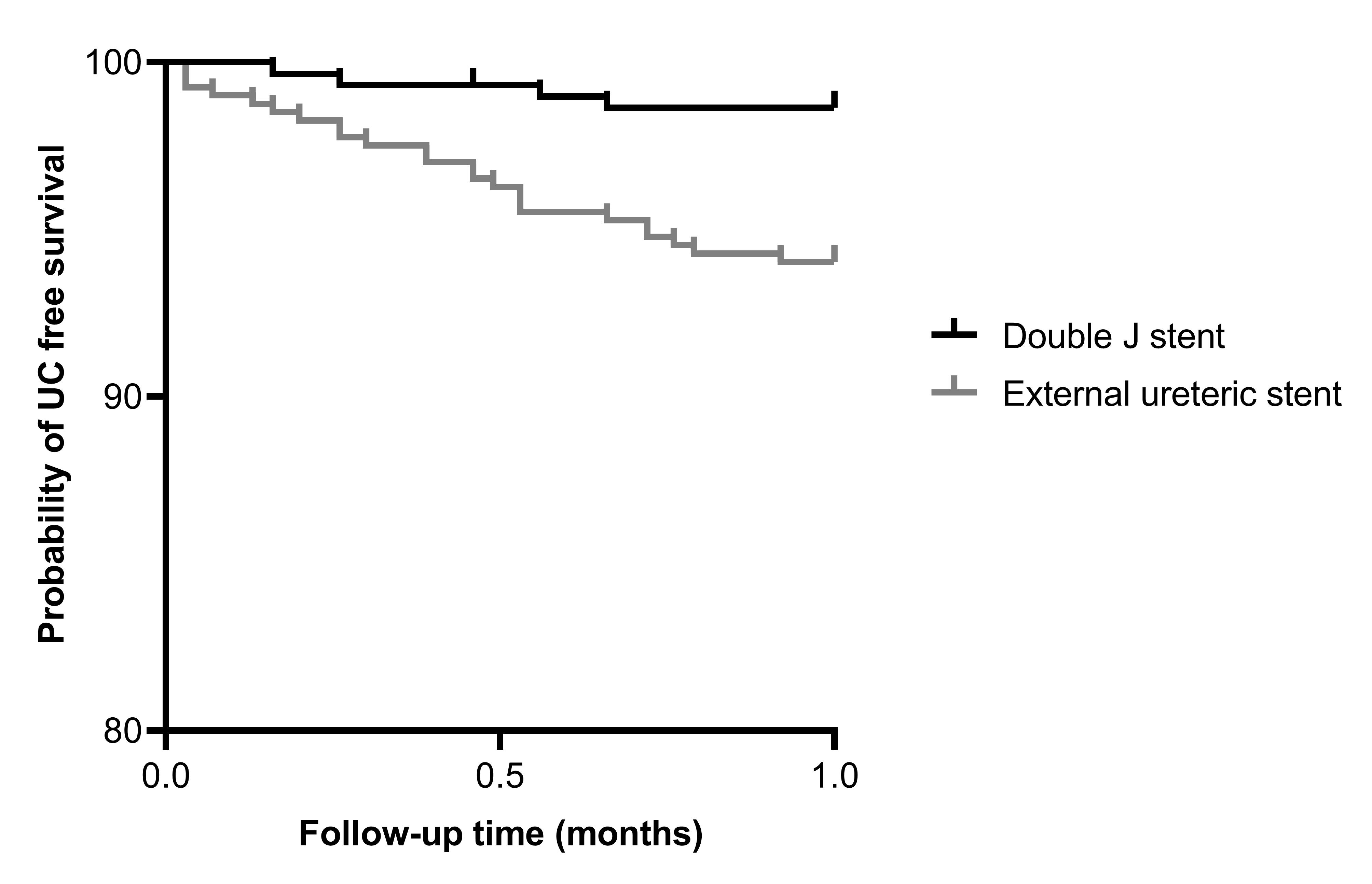

Supplement: Supplementary Figure 1 — Kaplan-Meier curve showing the probability of urological complication free survival within 1 month after kidney transplantation for external ureteric stent (n=403, UC n=24) and double J stent (n=294, UC n=4); HR 4.48 (95% CI 2.12-9.48), p=0.002. Four cases were censored before an urological complication occurred. [file Image_1.jpeg]

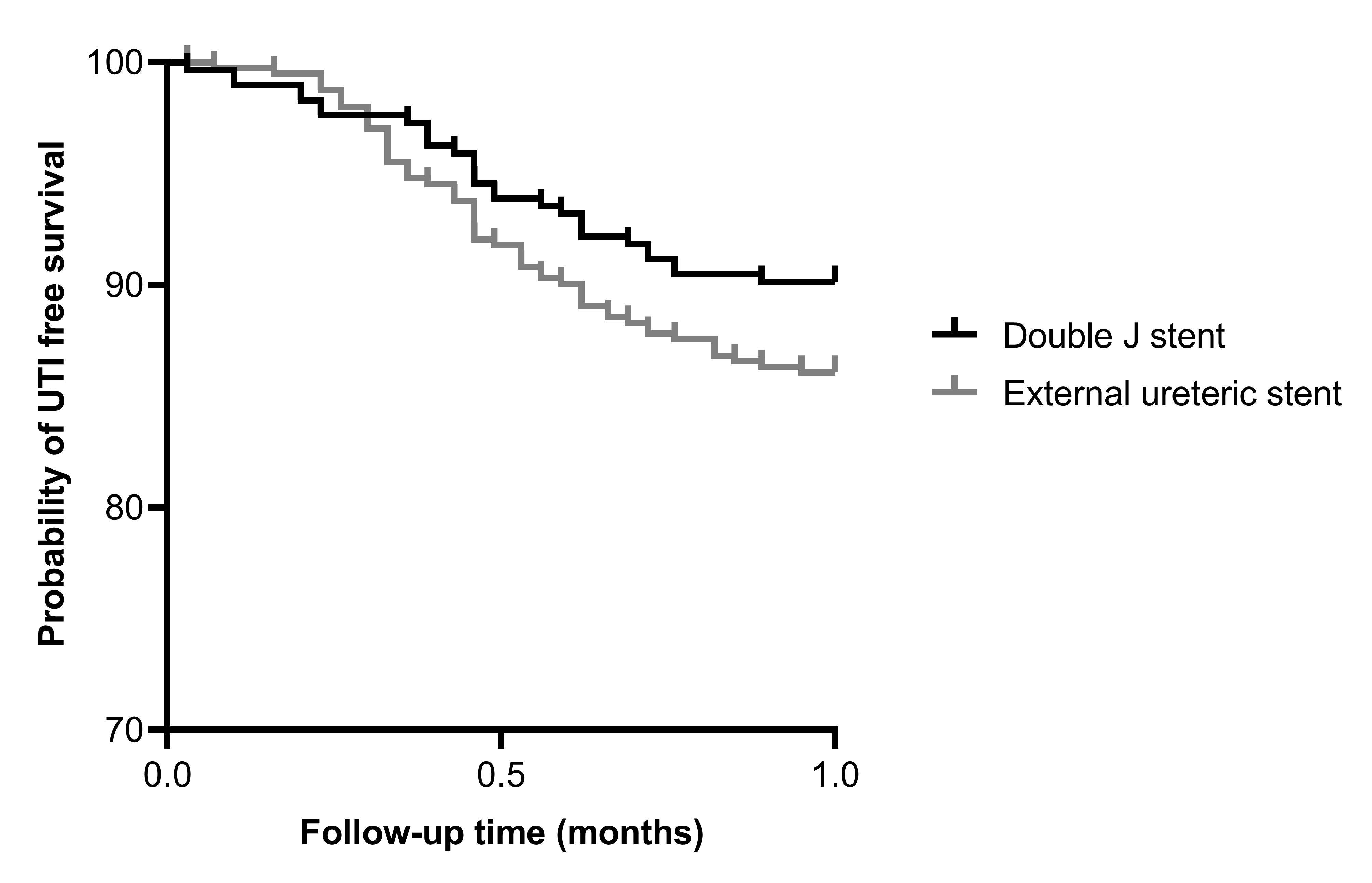

Supplement: Supplementary Figure 2 — Kaplan-Meier curve showing the probability of UTI free survival within 1 month after kidney transplantation for external ureteric stent (n=403, UTI n=56) and double J stent (n=294, UTI n=29); HR 1.44 (95% CI 0.94-2.21), p=0.109. Three cases were censored before a urinary tract infection occurred. [file Image_2.jpeg]
